# Cention N in non-carious cervical lesions: outcomes of a 24-month three-arm randomized controlled trial

**DOI:** 10.1186/s12903-026-09510-0

**Published:** 2026-08-03

**Authors:** Khattab Mustafa, Hussam Milly

**Affiliations:** 1 Faculty of Dentistry, Damascus University, Damascus, Syria; 2 Faculty of Philosophy and Social Sciences, University of Augsburg, Augsburg, Germany

**Keywords:** Cention N, Alkasites, Glass ionomer cements, Non-carious cervical lesions, Randomized controlled trial, Dental materials

## Abstract

**Objectives:**

This study evaluates the clinical performance of Cention N (CN), applied with either an adhesive or a retentive groove, in comparison with that of resin-modified glass ionomer cements (RM-GIC) in non-carious cervical lesions (NCCLs).

**Materials and methods:**

This three-arm, split-unit trial included 81 restorations (*n* = 27 per group): (1) RM-GIC (Fuji II LC); (2) Cention N with a universal adhesive (CN + UA); and (3) Cention N with a gingival retentive groove without adhesive (CN + RG). Clinical performance was assessed at 6, 12, and 24 months using modified USPHS criteria. Data were analysed using Generalized Estimating Equations (GEE), with Sequential-Bonferroni-adjusted *p*-values for pairwise comparisons (α = 0.05).

**Results:**

At 24 months, the recall rate was 77.8%. Retention rates were 100% for CN + UA, 95.2% for RM-GIC, and 61.9% for CN + RG. Significant differences in retention were observed between CN + RG and both CN + UA (*p* = 0.001) and RM-GIC (*p* = 0.013). Regarding marginal staining, significant differences emerged between the three groups at the 12- and 24-month intervals (*p* < 0.05).

**Conclusions:**

After 24 months of follow-up, CN applied with an adhesive demonstrated clinical performance comparable to RM-GIC. In contrast, CN placed without an adhesive system, despite the use of mechanical retention, exhibited significantly lower restoration retention.

**Clinical relevance:**

CN is a promising material for restoring NCCLs with high functional clinical performance. CN should be used with an adhesive system in NCCLs, as mechanical retentive features do not compensate for the absence of adhesive bonding.

**Trial registration:**

This trial was retrospectively registered at clinicaltrials.org, NCT05593159 (12.10.2022). https://clinicaltrials.gov/study/NCT05593159?cond=Cention&aggFilters=status:com%20unk&rank=2.

## Introduction

Secondary caries remain the primary cause of resin composite (RC) long-term restoration failure [1], driving continuous efforts to develop dental materials capable of reducing recurrent caries. In vitro studies show that material properties such as ion-release and bioactivity, defined as the ability to release sufficient ions to promote remineralization [2], may help reduce caries incidence by inhibiting bacterial activity, buffering acidic environments, and preventing tooth demineralization [3–6]. Glass ionomer cements (GICs) and resin-modified glass ionomer cements (RM-GICs) are well-established examples of such ion-releasing materials and have demonstrated low rates of secondary caries in both short- and long-term clinical studies [7]. In order to enhance the mechanical properties of conventional GICs, alternative restorative materials have been developed, including giomer-based materials such as Beautifil II and compomers such as Dyract, which have shown acceptable long-term performance [8, 9].

In recent years, newer restorative materials have been introduced, including advanced RM-GICs such as ACTIVA BioACTIVE and Surfil One, glass hybrid systems such as EQUIA Forte, and alkasite-based materials such as Cention N (CN) [2, 10]. Although these materials have shown promising clinical performance [11–13], several studies, including a recent meta-analysis evaluating the caries-preventive potential of bioactive materials, have reported no significant clinical advantage over conventional RC restorations in terms of secondary caries prevention and restoration retention [10, 14, 15]. However, most available clinical trials, including those included in this meta-analysis, are limited to short follow-up periods, which highlights the need for more studies with long observation periods to better assess the long-term clinical performance of these materials.

CN is a relatively recent addition to the restorative materials market, characterized as the first derivative of a composite resin with confirmed bioactivity [2, 16]. This dual-cure, bulk-fill restorative system is supplied in a powder-liquid format [2, 17]. The liquid component does not contain any water and comprises a blend of four monomers. The powder contains silane-treated fluoro-alumino-silicate (FAS) glass, unreactive fillers, and a unique alkaline glass filler, which classifies the material within the “Alkasite” family [16]. Current literature demonstrates that the clinical performance of CN in Class I and II restorations is comparable to that of conventional RC [12, 15, 18–22]. Additionally, studies show its efficacy in primary teeth as an alternative to GIC or stainless-steel crowns [23–27]. For non-carious cervical lesions (NCCLs), CN has emerged as a promising alternative to RM-GIC, which is considered the clinical standard restoration [28, 29]. However, clinical evidence remains sparse; while two randomized controlled trials reported comparable outcomes between CN and RM-GIC at 12 months, the longitudinal performance of CN in NCCLs beyond a one-year period has not yet been investigated [30, 31].

The retention of restorations in non-carious cervical lesions (NCCLs) is clinically challenging due to the inherently non-retentive geometry of these lesions [29]. Therefore, NCCLs are widely regarded as a rigorous clinical model for testing the retention of newly developed restorative materials. While the application of an adhesive system is mandatory for conventional RC, CN may be applied without an adhesive in retentive preparations [17]. Several studies have investigated the self-adhesive potential of CN, with one study reporting that the shear bond strength of CN without an adhesive (0.6 ± 1.0 MPa) was significantly lower than when an adhesive was utilized (8.3 ± 2.9 MPa) [32]. This suggests a markedly lower bond strength in the absence of an adhesive system. However, in vitro studies show that applying CN directly to the tooth structure facilitates its bioactive potential and the formation of hydroxyapatite at the tooth-restoration interface, while there is no evidence of CN bioactivity when an adhesive system is applied [16].

Previous research suggests that incorporating retentive grooves into NCCL preparations for RC restorations does not significantly enhance retention [33, 34]. This lack of benefit could be attributed to the superior bond strength provided by modern adhesive systems, which renders additional mechanical features redundant. However, in the case of (CN), such grooves may offer clinical utility, particularly when the material is utilized in an adhesive-free protocol. To date, no clinical studies have investigated whether these mechanical retentive features can effectively compensate for the absence of bonding by an adhesive system in CN restorations within NCCLs [35].

Clinical trials investigating the placement of retentive features in Class V cavities are scarce. This may partially be attributed to the ongoing shift toward minimally invasive dentistry and maximum preservation of dental tissues, which has reduced research interest in traditional mechanical retention strategies. As a result, the effectiveness of such retentive features remains insufficiently understood. Although the current prevalence of retentive feature utilization is difficult to estimate, studies indicate that dental practitioners in some regions continue to employ traditional restorative approaches, such as amalgam restorations, in clinical practice [36]. This may suggest the continued use of other conventional techniques, including mechanical retention features. Therefore, further research is warranted to evaluate the effectiveness and clinical relevance of interventions such as retentive features.

The clinical performance of CN warrants further investigation. To date, no investigations have examined its clinical performance in NCCLs for periods exceeding one year, or when it is applied without an adhesive and in combination with a retentive groove. Therefore, this study aims to evaluate the performance of CN compared to RM-GIC using two distinct protocols: application with a universal adhesive and application with a retentive groove and no adhesive. The null hypothesis states that there is no significant difference in retention between CN applied with an adhesive, CN applied with a retentive groove, and RM-GIC after 24-month follow-ups.

## Materials and methods

### Ethics approval and protocol registration

This study received ethical clearance from Damascus University (no./2777/2021). Prior to any restorative procedures, all participants provided written informed consent after a comprehensive explanation of the study’s procedures, aims, risks, and benefits. Participants were also informed that, should a restoration be lost or fail during the trial, they would be offered a replacement restoration. No formal adverse event assessment was undertaken. Harms were assessed non-systematically. The study report adhered to the CONSORT 2025 guidelines [37].

The protocol of this study was retrospectively registered at ClinicalTrials.gov (Clinical trial number: NCT05593159; Registration Date: 12.10.2022). Initially, the study was designed with a 12-month evaluation period. However, the research team elected to extend the evaluation to 24 months. This modification was implemented to contribute more significantly to the evidence regarding the long-term clinical performance of the materials. As no additional interventions were performed beyond routine follow-up examinations, approval for the extended observation period was obtained within the Department of Restorative Dentistry and Endodontics. Participants had consented to continued monitoring and follow-up according to the approved study procedures beyond the initial 12-month evaluation period.

The original study protocol was consistent with the protocol registered at ClinicalTrials.gov, and no modifications were made before registration. Prospective registration was not completed before participant recruitment due to time constraints associated with the academic timeline of the study. Registered outcomes related to gingival health have been reported previously in a separate publication [38]. Registered outcomes related to postoperative sensitivity were not included in the present report because they fall outside the scope of the current 24-month clinical evaluation.

### Study design, blinding and setting

This study was designed as a double-blind, three-armed, split-unit, randomized controlled trial. The clinical assessors and the patients were blinded to the restorative material used for each intervention. All clinical interventions and follow-up evaluations were performed at the Department of Restorative Dentistry, Faculty of Dentistry, Damascus University (Syria), between May 2022 and January 2025.

Clinical outcomes related to gingival health near the study restorations for 25 of the current participants were reported in a previous 9-month follow-up study [38]. The final sample of 27 in this report includes two patients enrolled between December 2022 and March 2023, whose data were not yet available at the time of the initial publication’s analysis.

### Sample size

The required sample size for this study was calculated to detect a significant difference in retention between three dependent groups over a two-year follow-up period. The expected proportions for the primary binary outcome at two years (P_CN+UA_ = 99.5%, P_CN+RG_ = 50.0%, and P_RM−GIC_ = 92.0%) were derived using a linear trend projection based on observed data from a one-year follow-up (P_CN+UA_ = 100%, P_CN+RG_ = 73.91%, and P_RM−GIC_ = 95.65%). Analysis of the one-year data indicated that the paired correlation between the groups was negligible and statistically non-significant; consequently, the sample size was determined using a conservative Chi-square framework. The calculated effect size (Cohen’s w) was 0.55. Using the R statistical software package (RRID: SCR_000432), with the significance level set at 0.05 and the desired statistical power at 0.90, it was determined that a minimum total sample size of 51 restorations (17 per group) is required to detect the specified differences.

### Recruitment and eligibility criteria

Patients were eligible for inclusion if they were at least 18 years of age and demonstrated good general and periodontal health, characterized by the absence of abnormal tooth mobility or deep periodontal pockets. Participants were required to have acceptable oral hygiene (minimum once-daily brushing and no generalized plaque) and at least 20 teeth in occlusion. Candidates required three or more non-carious cervical lesions (NCCLs), located on the labial/buccal surface of the tooth, exceeding 1 mm in depth, involving both enamel and dentin in vital teeth. The exclusion criteria were based on those used in two clinical studies evaluating NCCL restorations [39, 40] and included pregnancy, lactation, active severe bruxism, or xerostomia. Study participants were recruited from the patient population at the Faculty of Dentistry. Two calibrated restorative dentistry specialists performed the clinical screenings to assess candidate eligibility. The two recruiters did not have access to the random allocation sequence.

### Random sequence generation and allocation concealment

Within-subject randomization was performed by a researcher in the same department using Microsoft Excel (RRID: SCR_016137). Using the RAND function, random numbers were assigned to each tooth number in an adjacent column; the dataset was then sorted in ascending order by these generated values. Allocation was concealed from the operator through sequentially numbered, opaque, sealed envelopes, which were only opened upon the patient’s arrival. Treatment followed a standardized sequence, beginning with the tooth assigned the lowest number according to the Universal Numbering System and proceeding in a clockwise direction.

### Intervention

Baseline data regarding dietary habits and oral hygiene practices were collected to provide tailored oral health instruction and prevent traumatic habits. To ensure adequate oral hygiene and compliance, these recommendations were reinforced during every follow-up appointment, thereby minimizing confounding factors related to dental abrasion, abfraction, and erosion.

All operative procedures were conducted by a single operator. For procedural calibration, five restorations per study group were placed in accordance with the study protocol. These restorations were not included in the study. To ensure procedural consistency, the operator adhered to a standardized, written clinical protocol developed for this trial.

Prior to the restorative procedures, local anaesthesia was administered. The operative site was then cleaned with a fluoride-free pumice paste (ProphPaste Pro-N100, Switzerland) using a rubber cup under water cooling. To ensure proper isolation and marginal access, a retraction cord (Sure-cord, Korea) was gently placed using a universal gingival cord packer (Medesy, Italy). Final isolation was achieved via a rubber dam, utilizing B-clamp sets (Coltene/Whaledent, Inc., USA) to facilitate gingival tissue retraction.

Each participant received three restorations: one RM-GIC restoration, one CN with universal adhesive (CN + UA) restoration, and one CN with a Retentive Groove (CN + RG) restoration. The materials used, including their manufacturers, batch numbers, and compositions, are presented in Table 1. Regarding the CN + UA group, Tetric N-Bond Universal (TNU) (Ivoclar Vivadent, Liechtenstein) was applied in a one-step mode using a micro-brush and rubbed into the tooth surface for 20 s. The adhesive was then gently air-dried until a firm, glossy layer was achieved. Finally, light-curing was applied for 10 s at 1200 mW/cm^2^ using a Woodpecker LED-F curing light (Woodpecker Medical Instrument Co., Ltd., Guilin, China), which was calibrated prior to each use. For the CN + RG group, the lesion surface was gently roughened by using a round carbide bur (H1SEM.204.014 VPE5 or H1SEM.204.016 VPE 5, Komet Dental, Lemgo, Germany) on a low-speed handpiece. Subsequently, a fine gingival retentive groove was prepared approximately 0.5 mm from the dentin–enamel junction using a small round carbide bur (H1SEM.205 0.1 mm VPE 5, Komet Dental, Lemgo, Germany). The retentive groove depth was 0.2 mm. Preparations were done at 2000 rpm under low pressure without water cooling. To ensure cutting efficiency, each bur was discarded after five applications.

**Table 1:** Composition, manufacturer, and batch number of the materials used

Material	Manufacturer	Batch number	Composition
Cention N	Ivoclar-Vivadent, Liechtenstein	Z01V4K	Liquid: dimethacrylate, additive, initiator, and stabilizer. Powder: calcium fluoro-silicate glass, Ba-Al silicate glass, Ca-Ba-Al fluorosilicate glass, and ytterbium trifluoride
Tetric N bond Universal	Ivoclar-Vivadent, Liechtenstein	Z01C8G	HEMA, Bis-GMA, Ethanol, 10-MDP, Methacrylated carboxylic acid polymer, Camphorquinone, 2-dimethylaminoethyl methacrylate.
Fuji II LC	GC Corporation, Tokyo, Japan	Liquid 2101061 Powder 2107161	Liquid: HEMA, Polyacrylic acid, and water Powder: Fluoro-alumino-silicate glass
Dentin Conditioner	GC Corporation, Tokyo, Japan	2108271	20% polyacrylic acid

*HEMA* 2-Hydroxyethyl methacrylate, *Bis-GMA* Bisphenol A-glycidyl methacrylate, *10-MDP* 10-Methacryloyloxydecyl-Dihydrogen-Phosphate

Following pretreatment, CN (Ivoclar-Vivadent, Liechtenstein) was applied according to the manufacturer’s instructions. In the CN + RG group, the material was first adapted into the retentive grooves before the rest of the cavity was bulk-filled to restore the anatomical form (working time: 3 min). In the CN + UA group, the material was bulk-filled directly. All CN restorations were light-cured for 20 s at 1200 mW/cm^2^, with the curing light tip positioned perpendicularly and 1 mm away from the restoration surface.

Excess material was removed, and the restorations were finished using fine and extra-fine diamond burs (8852.314.014 and 852EF.314.014, Komet, Gebr. Brasseler GmbH & Co., Germany) under water cooling. Final polishing was achieved using Optrapol (Ivoclar Vivadent, Liechtenstein).

The RM-GIC restorations (Fuji II LC; GC, Tokyo, Japan) were placed according to the manufacturer’s instructions. After the cavities were washed and gently dried, Dentin Conditioner 20% (GC, Japan) was applied to the surface using a pellet and subsequently rinsed. Afterwards, for lesions with a depth of less than 2 mm, the Fuji II LC mixture was placed in a single bulk layer. For lesions exceeding 2 mm in depth, an incremental layering technique was employed, with each increment light-cured for 20 s. Consistent with the CN groups, finishing and polishing were performed in the same way.

### Calibration procedures for clinical evaluation

Two experienced dentists served as independent assessors for this study. Neither assessor participated in the initial patient eligibility screening nor the clinical restorative procedures. The assessors were blinded to the materials used and intervention procedures implemented in this trial and underwent standardized training by assessing the restoration-related parameters of 10 Class V restorations not included in the study sample. The restorations represented a variety of clinical conditions and were selected from cases presented at the Department of Restorative Dentistry and Endodontics.

### Clinical evaluation

Clinical performance was evaluated using the Modified United States Public Health Service (USPHS) criteria [39, 41, 42] (Table 2), defining restoration retention as the primary outcome. The restorations were evaluated at the baseline (one-week post-restoration) and at 6-, 12-, and 24-month follow-up intervals. The two assessors performed independent, blinded evaluations of all parameters. Following their individual assessments, the results were compared. In instances of disagreement, the assessors engaged in discussion to reach a consensus, ensuring a single unified data point for each parameter.

**Table 2:** Modified United States Public Health Service (USPHS)

Criteria	Alpha	Bravo	Charlie
Retention	Retained	Partially retained	Missing
Marginal staining	No discoloration along the margin	Slight and superficial staining	Deep staining cannot be polished away
Marginal adaptation	Restoration is continuous with the existing anatomic form	Detectable V-shaped defect in enamel only. Catches explorer going both ways	Detectable V-shaped defect to the dentin-enamel junction.
Fracture	None	Small chip, but clinically acceptable.	Failure due to bulk restorative fracture
Anatomic form	Appropriate contour	Slightly over/under contoured.	Unacceptably over/under contoured
Secondary caries	No evidence of caries contiguous with the margin	n/a	Evidence of presence of caries.
Patient satisfaction	Patient is totally satisfied	Patient has aesthetic complaints, or complaints related to the inability to chew comfortably.	Patient is completely unsatisfied

### Statistical analysis

Generalized Estimating Equations (GEE) were employed to compare the clinical performance of the three study groups at the 12- and 24-month follow-ups, regarding retention, marginal staining, and marginal adaptation. At each time point, all assessed restorations were included in the analysis. GEE, using an exchangeable working correlation matrix, was implemented to account for the correlated nature of the data within each participant and any missing data. To maintain the family-wise error rate across multiple comparisons, the sequential Bonferroni correction was applied to adjust *p*-values. A Fisher’s exact test was conducted to evaluate whether retention loss at the 6-month and 12-month follow-ups was associated with dropout at the 24-month follow-up. Statistical Package for Social Sciences (SPSS) software for Windows V31.0 (RRID: SCR_002865) was used to analyse the data.

## Results

**Fig. 1:**
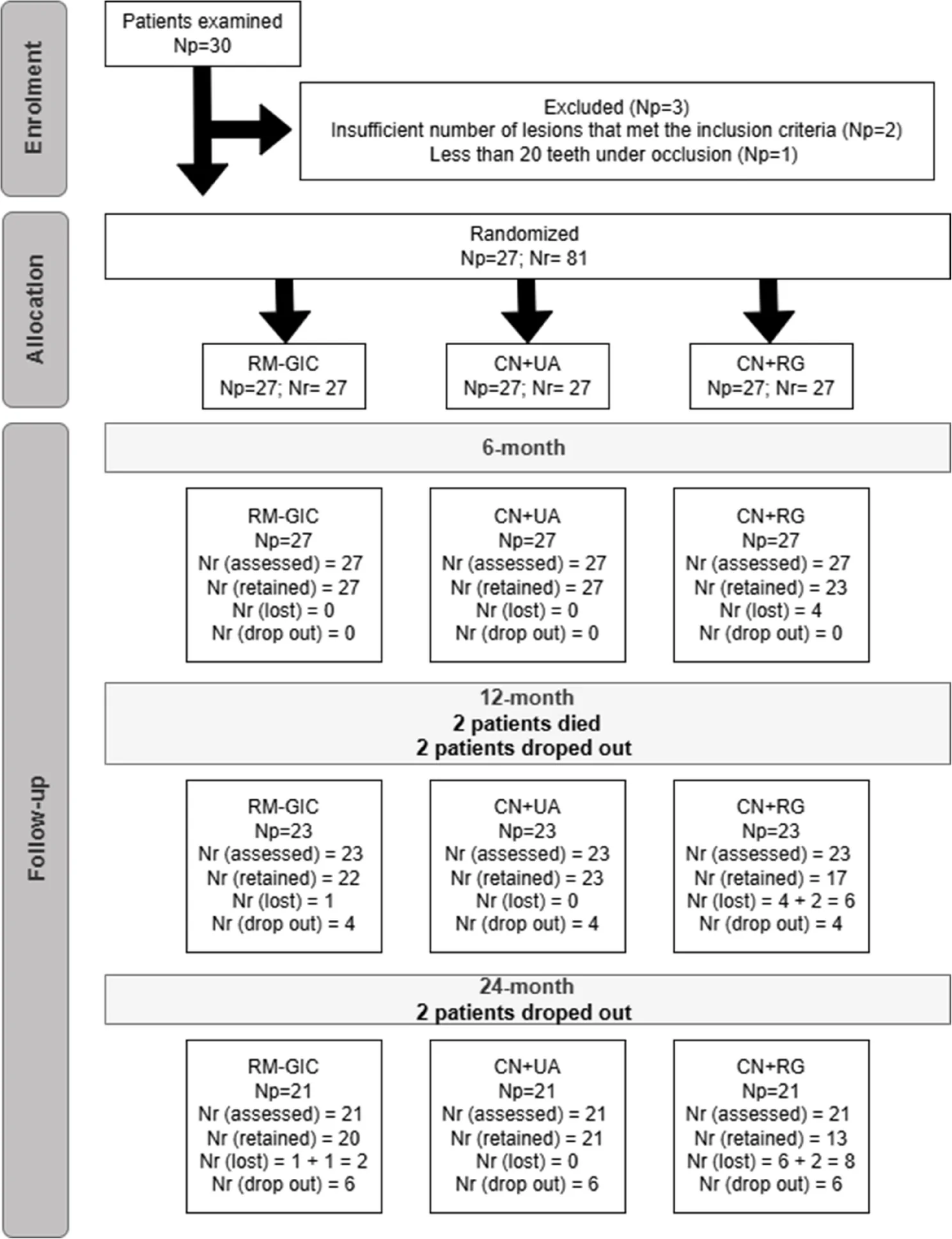
Flow diagram. Np, number of patients; Nr, number of restorations. RM-GIC = Resin-Modified Glass Ionomer Cement, CN + UA = Cention N with a universal adhesive, CN + RG = Cention N with a retentive groove

A total of 30 patients were screened for eligibility; following the application of inclusion and exclusion criteria, 27 participants were included in the final sample. Each subject received three restorations (one for each study arm), yielding 81 total restorations (Fig. 1). The single operator delivered all the restorations as intended. Detailed baseline subject and lesion characteristics are provided in Tables 3 and 4. Follow-up adherence was 100% at 6 months, declining to 85.2% and 77.8% at the 12- and 24-month intervals, respectively.

**Table 3:** Baseline characteristics of the patients included in the study

		*N*	%
Gender	Male	15	55.6
	Female	12	44.4
Parafunctional habits	Rarely/no	13	48.1
	Sometimes	9	33.3
	Often	5	18.5
Smoking	No	19	70.4
	Yes	8	29.6
Age	30–39	2	7.4
	40–49	5	18.5
	50–59	10	37
	60+	10	37

**Table 4:** Baseline characteristics of the teeth/lesions included in the study

	CN + UA *n* (%)	CN + RG *n* (%)	RM-GIC *n* (%)
Tooth type			
Canine	2 (7.4%)	2(7.4%)	4(14.8%)
Incisor	4 (14.8%)	6 (22.2%)	2 (7.4%)
Molar	4 (14.8%)	4 (14.8%)	4 (14.8%)
Premolar	17 (63.0%)	15 (55.6%)	17 (63.0%)
Tooth arch			
Maxillary	10 (37.0%)	10 (37.0%)	9 (33.3%)
Mandibular	17 (63.0%)	17 (63.0%)	18 (66.7%)
Attrition facets			
No	3 (11.1%)	5 (18.5%)	7 (25.9%)
Yes	24 (88.9%)	22 (81.5%)	20 (74.1%)
Dentin sclerosis			
0	1 (3.7%)	1 (3.8%)	1 (3.7%)
1	5 (18.5%)	4 (15.4%)	8 (29.6%)
2	16 (59.3%)	15 (57.7%)	9 (33.3%)
3	4 (14.8%)	5 (19.2%)	8 (29.6%)
4	1 (3.7%)	1 (3.7%)	1 (3.7%)
Cervico-incisal height			
< 1,5	1 (3.7%)	1 (3.7%)	3 (11.1%)
> 2,5	14 (51.9%)	16 (59.3%)	14 (51.9%)
1,5 − 2,5	12 (44.4%)	10 (37.0%)	10 (37.0%)
Shape (degree of angle)			
45–90	10 (37.0%)	8 (29.6%)	9 (33.3%)
90–135	15 (55.6%)	18 (66.7%)	16 (59.3%)
> 135	2 (7.4%)	1 (3.7%)	2 (7.4%)

Fisher’s Exact Test indicated no significant association between restoration loss and participant dropout (*p* = 1.000). Thus, restoration loss did not demonstrably increase the likelihood of study attrition.

### Retention

At the final 24-month evaluation, eight restorations in the CN + RG group had failed. In the RM-GIC group, one restoration had failed, along with one additional failure from a patient who dropped out of the study. There were no failures in the CN + UA group. While the groups performed comparably at 12 months (*p* = 0.083), the difference became statistically significant at 24 months (*p* = 0.035). Retention results are summarized in Table 5; Fig. 1.

**Table 5:** Clinical evaluation outcomes and the results of Wald Chi-square for between-group comparisons at different time-points

		CN + RG				CN + UA				RM-GIC				*p*-value^a^			
		T0	T1	T2	T3	T0	T1	T2	T3	T0	T1	T2	T3	T0	T1	T2	T3
		*n* (%)	*n* (%)	*n* (%)	*n* (%)	*n* (%)	*n* (%)	*n* (%)	*n* (%)	*n* (%)	*n* (%)	*n* (%)	*n* (%)				
Retention	Alpha	-	23 (85.2)	17 (73.9)	13 (61.9)	-	27 (100)	23 (100)	21 (100)	-	27 (100)	22 (95.7)	20 (95.2)	-	-^b^	0.083	0.035*
	Charlie	-	4 (14.8)	6 (26.1)	8 (38.1)	-	0	0	0	-	0	1 (4.3)	1 (4.8)				
Marginal staining	Alpha	27 (100)	19 (82.6)	10 (58.8)	8 (61.5)	27 (100)	27 (100)	21 (91.3)	19 (90.5)	27 (100)	27 (100)	18 (81.8)	14 (70)	-	-^b^	0.020*	0.005*
	Bravo	0	3 (13)	7 (41.2)	4 (30.8)	0	0	2 (8.7)	2 (9.5)	0	0	4 (18.2)	6 (30)				
	Charlie	0	1 (4.3)	0	1 (7.7)	0	0	0	0	0	0	0	0				
Marginal adaptation	Alpha	25 (92.6)	21 (91.3)	16 (94.1)	11 (84.6)	27 (100)	27 (100)	22 (95.7)	20 (95.2)	27	27	22 (100)	20 (95)	-	-^b^	0.826	0.516
	Bravo	2 (7.4)	2 (8.7)	1 (5.9)	2 (15.4)	0	0	1 (4.3)	1 (4.8)	0	0	0	1 (5)				
Fracture	Alpha	27 (100)	23 (100.0)	17 (100.0)	13 (100.0)	27 (100)	27 (100.0)	23 (100.0)	21 (100.0)	27 (100)	27 (100.0)	22 (100.0)	20 (100.0)	-	-	-	-
Anatomic Form	Alpha	27 (100)	23 (100.0)	17 (100.0)	13 (100.0)	27 (100)	27 (100.0)	23 (100.0)	21 (100.0)	27 (100)	27 (100.0)	22 (100.0)	20 (100.0)	-	-	-	-
Secondary caries	Alpha	27 (100)	23 (100.0)	17 (100.0)	13 (100.0)	27 (100)	27 (100.0)	23 (100.0)	21 (100.0)	27 (100)	27 (100.0)	22 (100.0)	20 (100.0)	-	-	-	-
Patient satisfaction	Alpha	25 (92.6)	23 (100.0)	17 (100.0)	13 (100.0)	27 (100)	27 (100.0)	23 (100.0)	21 (100.0)	27 (100)	27 (100.0)	22 (100.0)	20 (100.0)	-	-	-	-
	Bravo	2 (7.4)	0	0	0	0	0	0	0	0	0	0	0				

T0: Baseline, T1: 6 months follow-up, T2: 12 months follow-up, T3: 24 months follow-up

^a^ Wald chi-square

^b^ Undefined due to quasi-complete separation

* significant at the 0.05 level

At the 24-month follow-up, a significant difference in retention was observed between the RM-GIC and CN + RG groups, *p* = 0.013 (95% CI: 0.06–0.61), and between the CN + UA vs. CN + RG groups, *p* = 0.001 (95% CI: 0.13–0.63) (Table 6). The odds ratio for the difference between RM-GIC vs. CN + RG was 0.081 (95% CI: 0.008–0.835) (Table 7), indicating that RM-GIC restorations had significantly lower odds of retention loss compared to the CN + RG group. Nevertheless, because the CN + UA group experienced no failure events, the statistical model encountered quasi-complete separation, precluding a direct pairwise comparison with RM-GIC.

**Table 6:** Pairwise comparison of retention at 12 and 24 months

	T2			T3		
	95% CI		*p*-value^a^	95% CI	*p*-value^a^	
RM-GIC vs. CN + UA	-0.13–0.04	0.307		− 0.14–0.04		0.306
RM-GIC vs. CN + RG	-0.02–0.45	0.080		0.06–0.61		0.013*
CN + UA vs. CN + RG	0.04–0.48	0.013*		0.13–0.63		0.001*

T2: 12 months follow-up, T3: 24 months follow-up

^a^ Wald chi-square (Sequential Bonferroni adjustment)

* significant at the 0.05 level

**Table 7:** Odds ratios for between-group comparisons of retention and marginal staining at 12 and 24 months

	Follow-up	Comparison	Odd Ratio (95% CI)	*p*-value^a^
Retention	T2	RM-GIC vs. CN + UA	-^b^	-^b^
		RM-GIC vs. CN + RG	0.129 (0.013–1.303)	0.083
	T3	RM-GIC vs. CN + UA	-^b^	-^b^
		RM-GIC vs. CN + RG	0.081 (0.008–0.835)	0.035*
Marginal staining	T2	RM-GIC vs. CN + UA	0.415 (0.138–1.250)	0.118
		RM-GIC vs. CN + RG	3.215 (0.960–10.767)	0.058
	T3	RM-GIC vs. CN + UA	0.256 (0.057–1.156)	0.077
		RM-GIC vs. CN + RG	1.716 (0.499–5.904)	0.392

Reference group: RM-GIC

T2: 12 months follow-up, T3: 24 months follow-up

^a^ Wald chi-square

^b^ Undefined due to quasi-complete separation

* significant at the 0.05 level

### Marginal staining

At the 6-month follow-up, the CN + RG group presented three Bravo and one Charlie rating, whereas the CN + UA and RM-GIC groups maintained 100% Alpha ratings. By the 12-month interval, the frequency of Bravo scores increased to seven in the CN + RG group, two in the CN + UA group, and two in the RM-GIC group.

At the final 24-month evaluation, the CN + RG group exhibited four Bravo and one Charlie rating among the remaining restorations; notably, six restorations from this group that had previously scored Bravo or Charlie were lost at this stage. In the CN + UA and RM-GIC groups, Bravo ratings were observed in two and six restorations, respectively, with one previously Bravo-recorded restoration lost in the RM-GIC group by this stage.

Statistically significant differences were observed among the groups at both the 12-month (*p* = 0.02) and 24-month (*p* = 0.005) follow-ups. At the 12-month stage, pairwise comparisons indicated that the RM-GIC group showed a clinical trend toward superior performance compared to CN + RG; the difference was borderline significant (OR = 3.215; 95% CI: 0.960–10.767; *p* = 0.058). By the 24-month evaluation, the comparison between RM-GIC and CN + UA showed a trend toward superior performance in CN + UA; however, this trend did not reach statistical significance (OR = 0.256; 95% CI: 0.057–1.156; *p* = 0.077).

### Marginal adaptation

Throughout the study, the majority of restorations maintained an Alpha rating. By 24 months, four restorations were rated as Bravo (CN + RG: *n* = 2; CN + UA: *n* = 1; RM-GIC: *n* = 1). No statistically significant differences were observed between groups at the 12- or 24-month follow-up periods (*p* > 0.05) (Table 5).

### Fracture

The fracture rating of all restorations remained consistently at the Alpha level from baseline through the 24-month evaluation (Table 5).

### Anatomic form

The anatomic form of all restorations was consistently rated as Alpha from baseline through the 24-month assessment (Table 5).

### Secondary caries

All restorations achieved an Alpha score at all follow-up intervals (Table 5).

### Patient satisfaction

Most restorations in this study were rated as Alpha, except for two cases in the CN + RG group at the baseline (Table 5). These were scored as Bravo at the baseline evaluation following reports of mild tooth sensitivity, which spontaneously resolved by the time of the subsequent follow-up.

## Discussion

The mechanical and biological properties of CN have been examined in multiple in vitro studies. A recent network meta-analysis reported that CN exhibits physico-mechanical properties comparable to those of RC [43], while another investigation confirmed its bioactive behaviour [16]. These characteristics are consistent with the favourable clinical outcomes reported for CN in several clinical studies [12, 19, 20, 27, 30, 31, 38] and a recent meta-analysis of clinical studies [44]. However, no studies to date have evaluated its clinical performance in NCCLs beyond one year, nor when it is placed without an adhesive and in combination with a retentive groove. The present study was conducted as a split-unit, single-operator randomized controlled trial to address these gaps and to compare the clinical performance of CN with that of RM-GIC.

Regarding the primary criterion—retention—the results show a significant difference among the three groups at 24 months; thus, the main null hypothesis was rejected. The CN + UA group demonstrated the best clinical performance, with no restoration losses recorded throughout the study period. This outcome is likely attributable to the high bond strength provided by the universal adhesive TNU, as documented in multiple laboratory and clinical investigations [39, 45]. This adhesive contains the monomer 10-Methacryloyloxydecyl-Dihydrogen-Phosphate (MDP), which enhances bonding durability by forming stable collagen–MDP complexes through hydrogen bonding with dentinal collagen, while also generating MDP–Ca salts that precipitate onto the collagen scaffold [46]. These salts protect the collagen from degradation and contribute to a long-lasting adhesive interface [46]. The results of the present study align with two previous one-year investigations in NCCLs reporting retention rates of 92.9% and 82.5% for CN restorations placed with a universal adhesive [30, 31]. The higher retention observed in this study may also be related to strict adherence to the manufacturer’s instructions, which recommend applying TNU without prior etching. In contrast, the previous studies utilized selective-etch or etch-and-rinse techniques.

The CN + RG group exhibited the highest failure rates, with approximately 38% of restorations lost by the 24-month follow-up. The results indicate that this group had 92% higher odds of restoration loss compared with the standard material, RM GIC. This can primarily be attributed to the low shear bond strength of CN when placed without an adhesive system [32]. Additionally, parafunctional habits present in some patients may have further contributed to the high failure rate. Previous studies have shown that certain types of NCCLs are associated with parafunctional behaviours [47], as these habits could lead to the concentration of occlusion forces in the cervical area [48]. Although clinical evidence suggests that both GIC and RC restorations demonstrate a relatively acceptable retention in patients with parafunctional habits [49, 50], the CN + RG group may have been more vulnerable to these stresses due to the inherently low bond strength of CN when placed without an adhesive system. Consequently, the incorporation of a retentive groove might not have sufficiently compensated for the absence of adhesive bonding. These findings are consistent with results from a one-year clinical study on Class I and Class II CN restorations, which reported significantly higher retention rates when CN was applied with a universal adhesive compared to placement without an adhesive in Class II restorations [26].

The present study evaluated marginal adaptation and marginal staining. The CN + RG group demonstrated slightly more Bravo ratings for marginal adaptation than the other two groups across all four follow-ups; however, these differences were not statistically significant. A previous clinical study with a two-year follow-up on occlusal lesions reported comparable findings [15]. Regarding marginal staining, the CN + RG group exhibited three Bravo and one Charlie ratings at the 6-month follow-up, whereas no Bravo or Charlie ratings were observed in the other two groups until the 9-month follow-up. Notably, six restorations in the CN + RG group that had received earlier Bravo or Charlie ratings subsequently failed due to loss of retention by the 12-month or 24-month follow-ups. This observation might suggest a possible association between early marginal staining and subsequent retention failure; however, this should be interpreted cautiously, as it remains speculative and falls outside the main focus of the present study.

A significant difference in marginal staining was observed among the three groups at the 12- and 24-month follow-ups; however, no statistically significant differences were identified in the pairwise comparisons. Notably, the six restorations in the CN + RG group that initially received Bravo and Charlie ratings were therefore recorded as missing data following retention loss. Their exclusion from the analysis may have affected the statistical results.

The presence of multiple Bravo and Charlie ratings in the CN + RG group may be attributed to the reduced shear bond strength between CN and the tooth structure when the material is placed without an adhesive. In vitro studies have demonstrated significantly lower bond strength and greater microleakage when CN is applied without an adhesive compared with its application using an adhesive system [32, 51, 52]. The Bravo ratings observed in the CN + UA group may be explained by the relatively weak bond formed to enamel when using an ultra-mild low-pH universal adhesive in the one-step mode, which results in less effective enamel etching and consequently weaker adhesion [39, 53, 54]. Previous clinical findings have shown that CN restorations placed without an adhesive exhibit more marginal staining than those placed with an adhesive [20].

All restorations received an Alpha rating for the fracture and anatomic form criteria. This could be because the restorations in this study were placed for NCCLs and were not subjected to direct occlusal forces. These findings could also reflect the high wear resistance of the materials assessed.

No secondary caries were observed around any of the restorations throughout the study. Similar outcomes have been reported in two clinical investigations, where 31/31 and 23/24 CN restorations, respectively, showed no secondary caries at the two-year follow-up [12, 19]. The ion-releasing properties of the materials used—known to inhibit demineralization [16, 55]—together with CN’s ability to increase biofilm pH, influence Streptococcus mutans activity, and impede bacterial adhesion [56–58] may have contributed to the absence of secondary caries. Nevertheless, as this study did not assess variables related to biofilm behaviour or ion release, these explanations remain speculative.

Several recently developed ion-releasing materials claim to offer high clinical performance while reducing the risk of secondary caries; however, most available studies feature short- to medium-term follow-ups and rely on visual caries assessment [14], which can fail to detect up to 40% of lesions [59]. Fluorescence-based devices, such as the Quantitative Light-induced Fluorescence (QLF) devices, provide highly accurate assessments of caries, as supported by a recent meta-analysis [60]. Therefore, future research should evaluate the caries-preventive potential of modern ion-releasing materials using a combination of visual assessment and advanced digital diagnostic methods alongside extended follow-up periods.

Patients reported satisfaction with the restorations in all three groups throughout the study period. Two patients experienced mild tooth sensitivity and discomfort in the teeth restored without an adhesive system, one week after placement. This finding aligns with previous clinical studies that observed increased postoperative sensitivity when CN was used without an adhesive [21, 61]. In the present study, the sensitivity is likely attributable to tooth surface roughening and the preparation of the retentive groove. Overall, the generally positive patient perception of the restorations may be a reflection of their favourable clinical performance.

### Limitations and future research

The results of this study should be interpreted in light of several limitations. These include the relatively modest sample size and the loss to follow-up observed during the study period, both of which may have affected the precision and generalizability of the findings. In addition, the trial was registered retrospectively, and the follow-up period was extended from the originally planned 12 months to 24 months. Furthermore, as the study was conducted at a single center and all restorations were placed by a single operator, the generalizability of the findings to other clinical settings may be limited.

The sample size of this study was calculated using projections derived from one-year outcomes because no previous studies with a similar design and follow-up period were available. Although this approach has been used in previous studies [39], it represents a limitation as the projected retention rates may have influenced the precision of the sample size estimation.

While the split-mouth design of this study effectively controls for patient-related confounding factors (such as age, smoking, and parafunctional habits), baseline characteristics regarding cavity dimensions and tooth types varied slightly across the clinical groups. Although these variations reflect typical clinical scenarios, they represent a limitation that may have subtly influenced the retention of the restorations.

This trial compared CN only with RM-GIC, which represents a limitation of the study, particularly with respect to assessing the potential contribution of ion release to clinical performance and secondary caries prevention. As both materials are ion-releasing restorative materials, the present design does not allow the specific effects of ion release to be isolated or evaluated. Future long-term studies including a conventional resin composite comparator would provide more meaningful insights into the clinical relevance of the ion-release properties and the caries-preventive claims associated with these materials.

It is also important to note that due to quasi-complete separation, between-group comparisons at 6-month follow-up and between CN-UA and the other two groups at the 12- and 24-month follow-ups were not possible.

One focus of this study was to evaluate the clinical performance of CN when a retentive groove is made in NCCLs. The rationale was to test the material under conditions that challenge restoration retention. Although, in clinical practice, using an adhesive system would typically be more convenient than preparing a retentive groove, the latter can still represent a valuable option when the in vitro-observed bioactive properties of a restorative material are desired. For instance, in carious lesions following selective caries removal, preparing a retentive groove and placing CN without an adhesive could potentially help avoid further excavation toward the pulp while supporting remineralization of affected dentin. Therefore, future research investigating the clinical performance of CN in carious Class V lesions could incorporate mechanical retentive features. Such lesions, due to their shape and depth, may provide a more retentive cavity form, potentially resulting in higher retention rates while allowing clinicians to benefit from the in vitro-observed bioactive properties of CN.

## Conclusion

Based on the 24-month clinical results of this trial, the following conclusions can be drawn:

1. CN applied with a universal adhesive demonstrates clinical performance comparable to RM-GIC in the restoration of NCCLs.
2. The use of mechanical retention without an adhesive system for CN in NCCLs results in significantly inferior restoration retention.

## Clinical significance

Cention N shows high clinical performance for the restoration of NCCLs when used with a universal adhesive. The implementation of mechanical retentive features, such as gingival retentive grooves, does not provide sufficient retention to compensate for the absence of adhesive-based bonding in these lesions.

## Data Availability

The anonymized data are available as open data on Zenodo (10.5281/zenodo.19383803). The dataset includes tooth related characteristics, lesion characteristics, and the clinical evaluation parameters.

## Abbreviations

CN: Cention N
GEE: Generalized Estimating Equations
GIC: Glass ionomer cements
NCCLs: Non-carious cervical lesions
RC: Resin composite
RG: Retentive groove
RM-GIC: Resin‑modified glass ionomer cements
UA: Universal adhesive
